# Single-Cell Profiling Comparisons of Tumor Microenvironment between Primary Advanced Lung Adenocarcinomas and Brain Metastases and Machine Learning Algorithms in Predicting Immunotherapeutic Responses

**DOI:** 10.3390/biom13010185

**Published:** 2023-01-16

**Authors:** Yijun Wu, Kai Kang, Chang Han, Li Wang, Zhile Wang, Ailin Zhao

**Affiliations:** 1 Department of Thoracic Oncology, Cancer Center, and Laboratory of Clinical Cell Therapy, West China Hospital, Sichuan University, Chengdu 610041, China; 2 Department of Cancer Center, West China Hospital, Sichuan University, Chengdu 610041, China; 3 Department of Hematology, West China Hospital, Sichuan University, Chengdu 610041, China; 4 Department of Thoracic Surgery, West China Hospital, Sichuan University, Chengdu 610041, China

**Keywords:** lung adenocarcinoma, brain metastasis, single-cell sequencing, tumor microenvironment, machine learning

## Abstract

Brain metastasis (BM) occurs commonly in patients with lung adenocarcinomas. Limited evidence indicates safety and efficacy of immunotherapy for this metastatic tumor, though immune checkpoint blockade has become the front-line treatment for primary advanced non-small cell lung cancer. We aim to comprehensively compare tumor microenvironments (TME) between primary tumors (PT) and BM at single-cell resolution. Single-cell RNA transcriptomics from tumor samples of PT (N = 23) and BM (N = 16) and bulk sequencing data were analyzed to explore potential differences in immunotherapeutic efficacy between PT and BM of lung adenocarcinomas. Multiple machine learning algorithms were used to develop and validate models that predict responses to immunotherapy using the external cohorts. We found obviously less infiltration of immune cells in BM than PT, characterized specifically by deletion of anti-cancer CD8+ Trm cells and more dysfunctional CD8+ Tem cells in BM tumors. Meanwhile, macrophages and dendritic cells within BM demonstrated more pro-tumoral and anti-inflammatory effects, represented by distinct distribution and function of SPP1+ and C1Qs+ tumor-associated microphages, and inhibited antigen presentation capacity and HLA-I gene expression, respectively. Besides, we also found the lack of inflammatory-like CAFs and enrichment of pericytes within BM tumors, which may be critical factors in shaping inhibitory TME. Cell communication analysis further revealed mechanisms of the immunosuppressive effects associated with the activation of some unfavorable pathways, such as TGFβ signaling, highlighting the important roles of stromal cells in the anti-inflammatory microenvironment, especially specific pericytes. Furthermore, pericyte-related genes were identified to optimally predict immunotherapeutic responses by machine learning models with great predictive performance. Overall, various factors contribute to the immunosuppressive TME within BM tumors, represented by the lack of critical anti-cancer immune cells. Meanwhile, pericytes may help shape the TME and targeting the associated mechanisms may enhance immunotherapy efficacy for BM tumors in patients with lung adenocarcinomas.

## 1. Introduction

Lung cancer remains one of leading causes of cancer incidence and death, and there were more than 2.2 million new cases and 1.79 million deaths in 2021 worldwide [1]. Two major histological classifications of lung cancer are small cell lung carcinoma and non-small cell lung carcinoma (NSCLC), while the latter accounts for more than 80% of all cases [2]. Among NSCLC patients, lung adenocarcinoma (LUAD) is the most common subtype, presenting a high incidence with a proportion of more than 40% of lung cancers. Brain metastasis (BM) occurs frequently in LUAD patients, with its incidence ranging from 22% to 54% [3]. In spite of rapid advances in cancer management, LUAD with BM indicates a poor prognosis. Meanwhile, BMs are also the most common intracranial malignancies and lung cancer remains the primary disease in 40% to 50% of all patients [4,5].

For advanced LUAD, current therapeutics includes conventional chemoradiotherapy, targeted therapy and novel immunotherapy (immune checkpoint inhibitors, ICI). As the first-line treatment for epidermal growth factor receptor (EGFR)-mutated LUAD cases, tyrosine kinase inhibitors (TKIs) has achieved enormous success, and the third-generation TKI, osimertinib, specifically showed great efficacy on BM patients [6]. Furthermore, ICIs such as programmed death protein-1/programmed cell death-1 ligand 1 (PD-1/PD-L1) monoclonal antibody had been approved for the treatment of stage IV NSCLC, based on the expression level of PD-L1 on tumor cells. All these therapeutic advances require presentations of molecular profiling, and thus characterizing features of the tumor microenvironment (TME) may maximize the current treatment outcomes and inspire more thoughts on precision therapy.

Components within tumors includes not only cancer cells, but also a series of other types of cells such as immune and stromal cells and secreted regulative and effective molecules, all of which forms a complex TME. The traditional bulk sequencing averages out cell type-specific differences, while the single-cell RNA sequencing (scRNA-seq) technology can assess transcriptomes of each individual cell, providing more clear presentations of tumor ecosystems through a higher dimension. Several studies depicted the single-cell landscape of LUAD that identified the potential programs and mechanisms in cancer transformation and evolution [7,8,9,10]. However, none of them comprehensively revealed the comparative explorations between primary LUAD tumors (PT) and BM.

In this study, by integrating and analyzing scRNA-seq data that contained the largest number of BM specimens from LUAD patients, we comprehensively delineated distinct TME features between PT and BM and identified the potential immunosuppressive factors that may contribute to the unfavorable clinical outcomes of BM patients treated with ICI. Using external immunotherapy cohorts, we also developed and validated multiple machine learning models to optimally predict responses of BM tumors to the ICI treatment.

## 2. Methods

### 2.1. Collection of Human Specimens for LUAD and BM

All fresh tumor specimens in the study were biopsied from advanced LUAD patients by diagnostic procedures such as percutaneous puncture biopsy or bronchoscopy. All scRNA-seq data are available online in the Gene Expression Omnibus (GEO) database (https://www.ncbi.nlm.nih.gov/geo/, accessed on 1 March 2022) under the accession number of GSE148071, GSE131907, GSE186344 and GSE143423. All analyses in the study involving human subjects were in accordance with the Helsinki Declaration and the ethical standards of the Institutional Review Board of West China Hospital. The 3′ single-cell RNA sequencing (10x Genomics) was used to process cell suspensions.

### 2.2. Data Integration, Processing and Quality Control

Routine data integration of included datasets was performed using R package Seurat (version 4.1.0) (R Foundation for Statistical Computing, Vienna, Austria) [11]. Firstly, we eliminated genes expressed in fewer that ten cells. Secondly, cells that met all the following criteria could be picked out: (1) 500–10,000 genes identified; (2) 1000–20,000 unique molecular identifiers (UMI) detected; (3) mitochondrial reads <20%; (4) hemoglobin reads <1%. After quality control processes, the count data was standardly normalized to obtain the expression matrix for subsequent analyses. Then, SCTransform with 3000 variable features [12], Principle Component Analysis (PCA) and Harmony [13] were performed in sequence for reducing dimension and removing batching effects. Top one hundred principle components were used for construction of k-nearest neighbor (KNN) graph and Uniform Manifold Approximation and Projection (UMAP) embedding.

### 2.3. Trajectory Analysis

We applied Monocle2 (version 2.22.0) and Monocle3 (version 1.2.2) (Bioconductor, Boston, MS, USA) to determine the potential lineage differentiation trajectories of cell clusters [14,15]. In the subsequent analyses of signature scores, pseudotime of clusters of interest was integrated to investigate their developmental trends. The DDRtree method was applied for dimension reduction during the Monocle analyses. In Monocle 3, the similar normalization in Seurat was performed, and the mutual nearest neighbor method was used for the removal of batching effects [16].

### 2.4. Functional Enrichment Analysis

Marker genes were identified for each cell cluster using R package MAST (version 1.20.0) [17], with the proportion of cells that expressed certain gene ≥ 25%, the binary log of fold change (LogFC) ≥ 0.25 compared to other cells, and an adjusted *p* value < 0.05. Gene expression heatmap was plotted using the subset of marker genes with at least 25% difference in their fractions inside and outside each cluster, which represented the ratios of cells that expressed the genes among all cells inside and outside the cluster. The GO tool was used for enrichment analyses with R package clusterProfiler (version 4.2.2) [18]. For each cell cluster, the top five enriched biological process terms were displayed.

### 2.5. Gene Signature Scores

To better compare phenotypes of cell clusters, we calculated certain signature scores based on specific gene expressions. T cell exhaustion scores were obtained based on differential marker genes between exhausted and non-exhausted CD4+ and CD8+ cells, with the FC larger than 1.5 and a minimal difference of 25% in the fraction of cells that expressed these genes. The naiveness score involved four canonical marker genes, including *TCF7*, *CCR7*, *SELL* and *LEF1*. Besides, other signature scores were defined based on previous literatures, involving regulatory T cell (Treg) [19], cytotoxicity [20] and proliferation [21] for T/natural killer (NK) cells, classical activation, alternative activated, angiogenesis and phagocytosis for tumor-associated macrophages (TAM) [22], and activation, migration and tolerance for dendritic cells (DC) [22,23]. Furthermore, R package AUCell (version 1.16.0) was applied to calculate whether a key subset of the input gene sets was enriched within each cell’s expressed genes, and presented a signature score for the subset [24]. In analyses of relationship between pseudotime and signature scores, general additive models were used for fitting.

### 2.6. Copy Number Variations and Tumor Heterogeneity

Copy number alterations (CNA) were assessed for epithelial cells with R package inferCNV (version 1.10.1) [25]. A total of 2000 immune cells were randomly obtained for reference and the cutoff value of minimal average reads per gene among reference cells was set as 0.1. The Vald D2 method was used for denoising and hierarchical clustering.

We calculated heterogeneity scores based on principle components of the gene expression matrix to denoise, and the principle components during the dimension reduction and clustering were identified as features of cancer cells. The calculation formula was as follows, and it can output the average Euclidean distance of each cell from the centroid in Euclidean space:

$$Heterogeneity\;score=\frac{1}{m}\overset{m}{\underset{i=1}{\sum }}\sqrt{\overset{n}{\underset{j=1}{\sum }}{\left(x_{ij}-y_{j}\right)}^{2}}$$

In this formula, *m* represents the number of tumor cells and *n* indicates *n* features in each cell. *x_ij_* represents the *j*-th feature of *i*-th cell. For intratumoral heterogeneity, *y_j_* represents the arithmetic average value of the *j*-th feature of all cells, while for intertumoral heterogeneity, *y_j_* represents the arithmetic average value of the *j*-th feature of cells in the BM or PT group. Z-score normalization of the scores was performed for distribution consistency.

### 2.7. Transcription Factor Analysis

To identify activated transcription factors (TF) within one cell cluster, we applied R package SCENIC (version 1.2.4) and python package pySCENIC (version 0.11.2) for TF analyses. Firstly, using the GRNBoost2 function in pySCENIC, we established a co-expression network to find potential targets for TFs. RcisTarget was used for enrichment analyses of TF motifs (reference: 500bp-upstream-7species.mc9nr, from cisTarget database, https://resources.aertslab.org/cistarget/, accessed on 1 April 2022), and AUCell was used to quantify regulons. A binary matrix could be created with the cutoff obtained by AUC scoring for each regulon. In TF analyses, the significant TF was defined as more than 20% of cells activated with it within each cell cluster (except for those in stromal cells, for which 50% was set as the cutoff).

### 2.8. External Validation in Bulk RNA Sequencing

A total of 526 LUAD patients from The Cancer Genome Atlas (TCGA) (https://portal.gdc.cancer.gov/, accessed on 15 April 2022) database were used to validate relationships between survival outcomes and signature scores of marker genes with differential expressions in the inflammatory-like cancer-associated fibroblasts (iCAF) and myofibroblast CAF (myCAF) clusters from the scRNA-seq data. The signature score was calculated using R package GSVA (version 1.42.0), with the single-sample gene set enrichment analysis (ssGSEA) selected [26]. For each cell cluster, the top 30 significant marker genes were as the reference list. In the survival analyses, all cases were separated into two groups by the median score. Survival curves were plotted using the Kaplan–Meier method and compared by the log-rank test.

### 2.9. Cell-Cell Interaction Analysis

Cell-cell interaction analyses were performed using Python package CellPhoneDB (version 3.0.0) (Teichmann Lab and the Vento-Tormo Lab (Wellcome Sanger Institute, Cambridge, UK)). The proportion of cells expressing each gene and the gene’s average expression level within a cell cluster were evaluated. Then the significance for each ligand-receptor pair was assessed based on their respective expression level, and the significant pairs for at least one cell–cell pair were retained.

### 2.10. Statistical Analysis

Statistical analyses were performed with R software (version 4.1.2, https://www.r-project.org). The comparisons between groups were conducted using Student’s *t* test, a chi-square test and Wilcoxon rank-sum test. In the correlation analyses, Pearson and Spearman methods were used. Machine learning algorithms including adaptive boosting (ADB), artificial neural network (ANN), gradient boosting decision tree (GBDT), random forest classifier (RFC) and extreme gradient boosting (XGB) and KNN were used to develop multiple models predicting responses to ICI therapy. Before modeling, the least absolute shrinkage and selection operator (LASSO) regression was performed for gene selection. Predictive performance for each model was measured by the area under the receiver operating characteristic (ROC) curve (AUC). To try to avoid data bias and improve the machine learning models’ adaptability, the most matched combination of two test datasets was identified to calculate the testing AUC value for each algorithm. *p* value < 0.05 was considered statistically significant in this study.

## 3. Results

### 3.1. The Global Single-Cell Landscape of Primary LUAD and Brain Metastases

**Figure 1:**
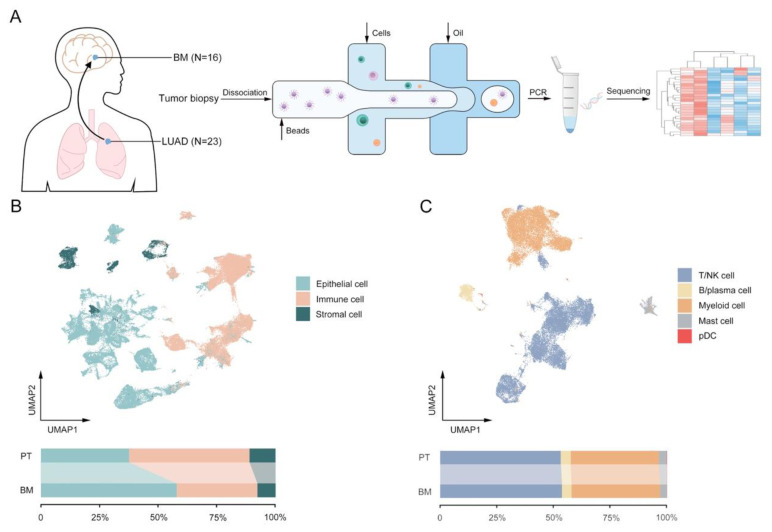
Single-cell landscape in primary tumors (PT) and brain metastases (BM) in patients with lung adenocarcinomas (LUAD). (**A**) Flowchart of single-cell transcriptomics data from human samples. (**B**) UMAP plot and proportions of epithelial, stromal and immune cells. (**C**) UMAP plot and proportions of subclusters of immune cells: T/natural killer (NK), B/plasma, myeloid, mast and plasmacytoid dendritic cells (pDC).

To elucidate the dynamic landscape and heterogeneity in the progression of LUAD to brain metastasis, fresh human tissue samples of twenty-three primary LUAD tumors (P1–P23) and sixteen brain metastases (M1–M16) were biopsied for scRNA-seq analyses (Figure 1A). Through quality filtering, a total of 106,605 cells were detected with a mean number of 2116 genes per cell, among which, 44,251 and 62,354 cells came from PT and BM, respectively (Figure S1A). Via annotations with canonical marker gene expression, several major cell lineages were identified, including epithelial (*EPCAM*), immune (*PTPRC*), stromal (*PECAM1*, *COL1A1*) and other cells (Figure 1B and Figure S1B). Furthermore, immune cells were clustered into T lymphocytes/natural killers (T/NK; *CD3D*, *KLRD1*), B lymphocytes/plasma (*CD79A*, *MZB1*), myeloid (*CD14*, *CD1C*), mast (*CPA3*) and plasmacytoid dendritic cells (pDC; LILRA4, CLEC4C) (Figure 1C and Figure S1C). As expected, though collected from different patients, almost all of immune and stromal cells clustered together by different subtypes, while cancer epithelial cells demonstrated greater heterogeneity between PT and BM in spite of obvious inter-patient disparities in transcriptomic signatures (Figure S2A,B) [9,27]. Similar to the previous studies, comparisons of the cell number between the two groups indicated a higher proportion of immune and stromal cells in PT than BM (Figure 1B), predominantly supported by more filtrations of T/NK (2051 vs. 1548 per 10,000 cells) and myeloid cells (1501 vs. 1126 per 10,000 cells) cells (Figure 1C and Figure S2E) [8]. Nevertheless, for both PT and BM, the proportions of all cell types varied vastly between tumor samples, suggesting the necessity to further decipher the underlying differences between tumor ecosystems of primary LUAD and BM (Figure S2C,D).

### 3.2. Deletion of CD8+ Trm Cells and More Dysfunctional CD8+ Tem Cells within BM Tumors

**Figure 2:**
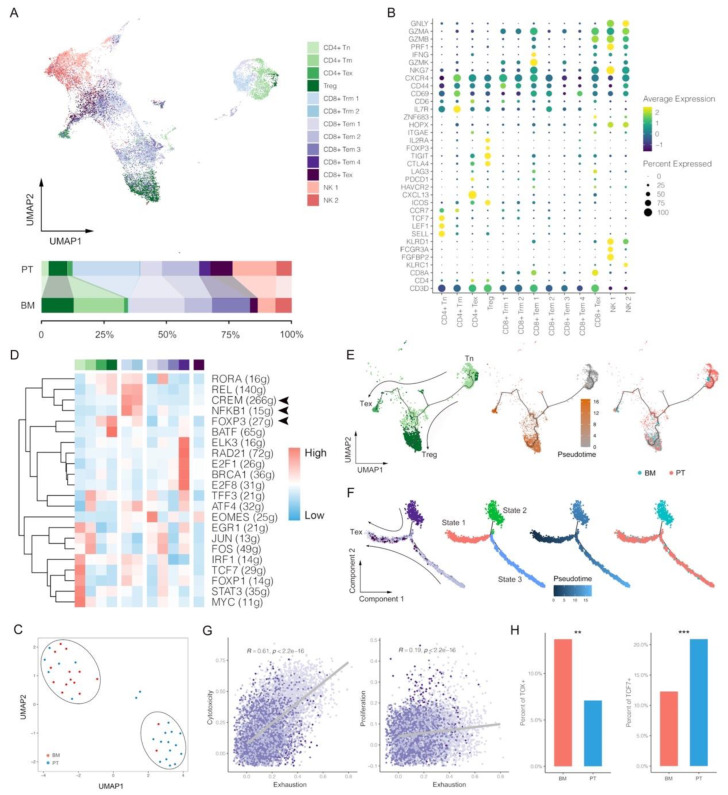
Single-cell characterization of T/natural killer (NK) cells in primary tumors (PT) and brain metastases (BM) of lung adenocarcinomas. (**A**) UMAP plot and proportions of T/NK clusters. (**B**) Average expression and cell precent expressed of marker genes in each T/NK cluster. (**C**) Clustering of T/NK proportion features between PT and BM groups. (**D**) Normalized activations of transcription factors in each T/NK clusters. The number of predicted target genes for each transcription factor was shown in the latter brackets. (**E**,**F**) Pseudotime trajectory analysis of CD4+ and CD8+ T lymphocytes. (**G**) Correlations between exhaustion and cytotoxicity/proliferation scores of CD8+ Tem. (**H**) Proportions of TOX+ or TCF7+ cells in CD8+ Tem between BM and PT (Chi-square test; ** *p* value < 0.01, *** *p* value < 0.001). CD4+ naïve T cell (CD4+ Tn), CD4+ memory T cell (CD4+ Tm), CD4+ exhausted T cell (CD4+ Tex), Treg, CD8+ tissue-resident memory T cell (CD8+ Trm), CD8+ effector memory T cell (CD8+ Tem), CD8+ exhausted T cell (CD8+ Tex).

Since T/NK cells were found more in primary lesions of LUAD than BM, we then further divided them into thirteen subclusters of three major classes (CD4+ T, CD8+ T and NK cells), involving CD4+ naïve T cell (CD4+ Tn), CD4+ memory T cell (CD4+ Tm), CD4+ exhausted T cell (CD4+ Tex), Treg, CD8+ tissue-resident memory T cell (CD8+ Trm), CD8+ effector memory T cell (CD8+ Tem), CD8+ exhausted T cell (CD8+ Tex) and NK cell (Figure 2A), identified with the corresponding canonical gene markers (Figure 2B). Based on T/NK cell proportion features, all patients can be greatly clustered into two groups that were extraordinarily consistent with tumor groups, suggesting infiltration differences of major anti-cancer immune forces between PT and BM (Figure 2C). Firstly, as for T cells, distribution analyses of subclusters demonstrated that CD4+ Tn, CD8+ Trm and CD8+ Tex were obviously higher in the PT group, while Treg and CD4+ Tm higher in the BM group. The lack of CD8+ Trm in BM may be associated with lower responses to immune checkpoint inhibitors (ICI) since previous studies showed a series of suppressive immune checkpoints highly expressed in CD8+ Trm cells [28,29]. Besides, the GO analysis of CD8+ T clusters also revealed that the T cell activation pathway was enriched in Trm rather than Tem (Figure S3A). Thus, within the TME of BM, CD8+ Tem that showed less expected anti-tumor ability accounted for the majority of T cell infiltrations, partly explaining the poor survival outcome of BM patients. We hypothesize that BM presents a dysfunctional anti-cancer immune phenotype caused by lacking main anti-cancer immune forces of CD8+ Trm cells.

TF analyses for each T cluster identified CREM and NFKB1 activated specifically in CD8+ Trm (Figure 2D). CREM was found to be correlated with immune-suppressive microenvironment, thus partly explaining the greater anti-cancer efficacy of CD8+ Trm by ICIs [30]. Additionally, the activation of NFKB1 also indicated the construction of a pro-inflammatory microenvironment by CD8+ Trm. As expected, FOXP3 predominantly activated in Treg and CD4+ Tex, and within each tumor, whether for PT or BM, the percentage of them showed significant correlation (Pearson correlation, R = 0.45, *p* = 0.005; Figure S3B).

We then performed pseudotime analyses of CD4+ and CD8+ T lymphocytes to reveal potential developmental trajectories of their subsets. In CD4+ T, two branches of Treg and Tex were observed from the same developmental origin of Tn, indicating their different gene expression profiles after differentiation (Figure 2E). However, the result of CD8+ T showed a consistent direction as pseudotime, along which all CD8+ T cell populations of state 1 (mainly Tem) and state 2 (Tem and Trm) developed into exhaustion (Figure 2F). Thus, we infer that CD8+ Tem and Trm have no intersection in the development but would come to a same exhausted end. Within the primary LUAD, CD8+ Tex can come from both CD8+ Trm and Tem [21]; however, while within the BM tumor, it may be predominantly transformed from CD8+ Tem rather than Trm.

The signature scores were assessed for each T subcluster, involving exhaustion, proliferation, naiveness, Treg (for CD4+ T) and cytotoxicity (for CD8+ T), which was consistent with the clustering result of T lymphocytes (Figure S3C). For example, Tn and Tex had highest naiveness and exhaustion scores, respectively. Similar to the previous observations, CD8+ Tex cells were also found to have much higher cytotoxicity score because they had been activated before exhaustion [31]. Then we compared the scores between PT and BM, and found that proliferation and naiveness scores for CD4+ T were significantly higher in BM than PT, but the BM group had a higher Treg scores, indicating the more inhibitory effects within the TME of BM (Figure S3D). Because of lack of CD8+ Trm in BM, we therefore compared scores of CD8+ Tem between PT and BM, and found that CD8+ Tem demonstrated significantly higher cytotoxicity and proliferation scores but also had a higher exhaustion score in BM (Figure S3E). Thus, we hypothesize that in spite of the infiltration of CD8+ Tem cells in BM, they may be more likely in transformation to Tex with expressions of certain exhaustion gene signatures (as CD8+ Tem 1 that mainly existed in BM, part of which highly expressed *TIGIT*, *CTLA4*, *LAG3*, *PDCD1* and *HAVCR2*; Figure 2B). This result was further consolidated by the significant positive correlations of cytotoxicity with exhaustion (Pearson correlation, R = 0.61, *p* < 0.001) and proliferation (Pearson correlation, R = 0.19, *p* < 0.001) scores in CD8+ Tem (Figure 2G). Meanwhile, exhaustion scores in CD8+ Tem also sharply increased as pseudotime (R = 0.66, *p* < 0.001; Figure S3F), and the BM group demonstrated a higher proportion of Tem cells expressing *TOX*, a gene encoding an important TF for T cell exhaustion, further indicating a more exhausted state of Tem population in BM (Figure 2H) [32]. Moreover, we also observed a significant lower percentage of CD8+ Tem cells expressing *TCF7* in BM than PT, a gene encoding TCF-1 that was critical to maintain long-term responses to ICI (Figure 2H). Overall, we found there were fewer filtrations of T lymphocytes in BM, especially lack of CD8+ Trm cells, which were critical to anti-cancer immunity in primary LUAD [10]. Meanwhile, CD8+ Tem in BM also showed more exhausted features than PT. These results may indicate potential mechanisms by which PT and BM present heterogeneous responses to ICI.

NK cells were further clustered into six subgroups based on the signature gene expressions, representing two classifications of CD16^dim^ and CD16^bright^ (Figure S3G,H). CD16, also called FcγRIII, is located at the surface of cells, which can initial antibody-dependent cellular cytotoxicity (ADCC) to activate NK after binding to the corresponding immunoglobulin G (IgG) [33]. We found a lower percentage of CD16^bright^ NK cells that highly expressed FcγRIII in BM, indicating a less functional NK distribution within the BM tumor. Besides, though with the higher NK activation score, the BM group demonstrated higher inhibition and lower cytotoxicity scores (Figure S3I). These results may suggest NK cells within the BM tumor have a more dysfunctional or exhausted phenotype than PT.

### 3.3. Macrophages and DCs within BM Tumors Reveal More Pro-Tumoral and Anti-Inflammatory Effects

**Figure 3:**
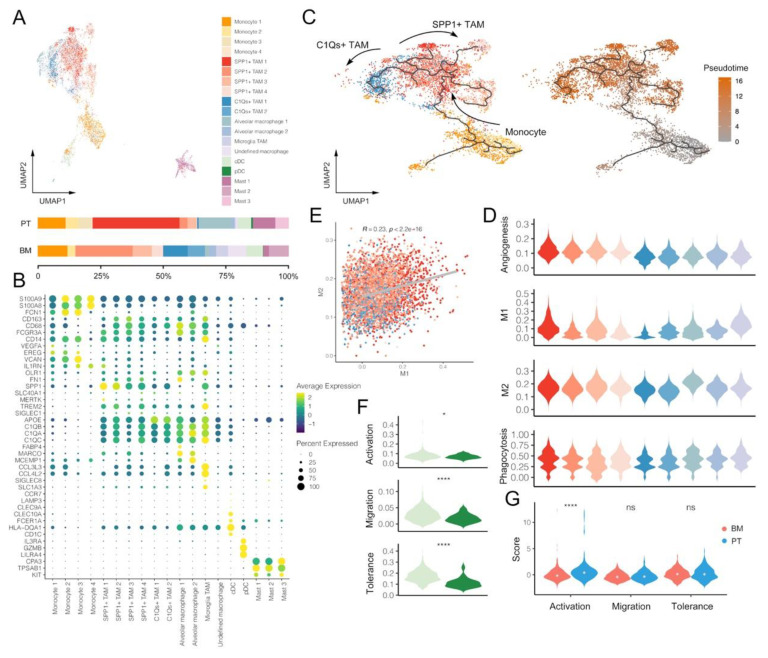
Single-cell characterization of myeloid cells in primary tumors (PT) and brain metastases (BM) of lung adenocarcinomas. (**A**) UMAP plot and proportions of myeloid cell clusters. (**B**) Average expression and cell precent expressed of marker genes in each myeloid cell cluster. (**C**) Pseudotime trajectory analysis of monocytes and TAMs. (**D**) Signature scores of gene sets associated with M1, M2, angiogenesis and phagocytosis in each TAM cluster. (**E**) Correlation between M1 and M2 scores of TAMs (Pearson correlation). (**F**) Signature scores of gene sets associated with activation, migration and tolerance between cDC and pDC (Wilcox rank-sum test; * *p* value < 0.05, **** *p* value < 0.0001). (**G**) Comparison of DC activation, migration and tolerance scores between PT and BM groups (Wilcox rank-sum test; **** *p* value < 0.0001). Tumor-associated macrophages (TAM).

Myeloid cells were clustered into five major classifications of monocytes, TAMs, other macrophages, DC and mast cells based on the corresponding gene markers (Figure 3A,B and Figure S4A). We identified two subtypes of TAMs with different phenotypes, including SPP1+ TAM (marked by *SPP1*, *FN1*, etc.) and C1Qs+ TAM (marked by *C1QB*, *C1QA*, *C1QC*, *TREM2*, *APOE*, etc.). C1Qs+ TAM highly expressed complement C1q-related genes, which could promote phagocytosis and anti-inflammatory function within TME [34]. Although both groups of tumors involved SPP1+ and C1Qs+ TAMs, their distributions were found to be specific to the tumor origins. For example, SPP1+ TAM1 was only observed in primary LUAD, while SPP1+ TAM2 and TAM3 mainly existed in BM. At the same time, there were much fewer C1Qs+ TAMs in PT than BM, suggesting a more disadvantageous immune-inhibited microenvironment within BM tumors. To better investigate the relationship between SPP1+ and C1Qs+ TAM, we performed pseudotime analyses of monocytes and TAMs, revealing that both of them might originate from monocytes, but through two distinct developmental trajectories (Figure 3C).

We then assessed signature scores that associated with macrophages for each TAM subcluster. Firstly, TAMs could be divided to two extreme situations in vitro, M1 and M2 that represented pro- and anti-inflammatory phenotypes, respectively. However, our results demonstrated C1Qs+ TAM clusters had lower M1 and M2 scores at the same time, while SPP1+ TAM clusters had relatively higher M1 and M2 scores (Figure 3D). Indeed, a significantly positive correlation was observed between M1 and M2 scores among TAMs, thus further suggesting that the polarization of TAM into M1 and M2 might be overemphasized, and there might be more complex mechanisms under these distinct TAM phenotypes (Pearson correlation, R = 0.23, *p* < 0.001; Figure 3E). Besides, SPP1+ TAMs were also found to have obviously higher angiogenesis scores, highlighting its complex roles in shaping TME.

Additionally, we identified several specific types of macrophages, such as alveolar macrophages (marked by *MARCO* and *MCEMP1*) and microglia TAMs (marked by *SLC1A3*, *SIGLEC8*, *CCL4L2* and *CCL3L3*) (Figure 3B and Figure S4A), which were specifically located at tumors of primary LUAD and BM, respectively (Figure 3A) [7,10,31]. Alveolar macrophage 1 demonstrated a much higher M1 score, suggesting its pro-inflammatory effects in PT (Figure 3D). By contrast, microglia TAMs might have both pro-inflammatory and anti-inflammatory phenotypes with similar average M1 and M2 scores. These results may be helpful in explaining the TME heterogeneity shaped by macrophages between PT and BM.

Two clusters of DCs were identified, including classical DC (cDC; marked by *CD1C*, *HLA-DQA1*, *FCER1A* and *CLEC10A*) and plasma DC (pDC; marked by *LILRA4*, *IL3RA* and *GZMB*) (Figure 3B). A similar distribution of cDC and pDC was observed between PT and BM. Therefore, we further compared their signature scores and found cDC demonstrated higher scores of all related functions that involved activation, migration and tolerance (Figure 3F), and the activation score was significantly correlated with both migration and tolerance scores (Figure S4B). Moreover, the DC activation score was significantly lower in the BM group than PT, suggesting the more inhibited antigen presentation capacity in DC cells of BM tumors (Figure 3G).

### 3.4. Cancer Epithelial Cells in BM Demonstrate Limited Heterogeneity and Lower Expression Levels of HLA-I-Associated Genes

**Figure 4:**
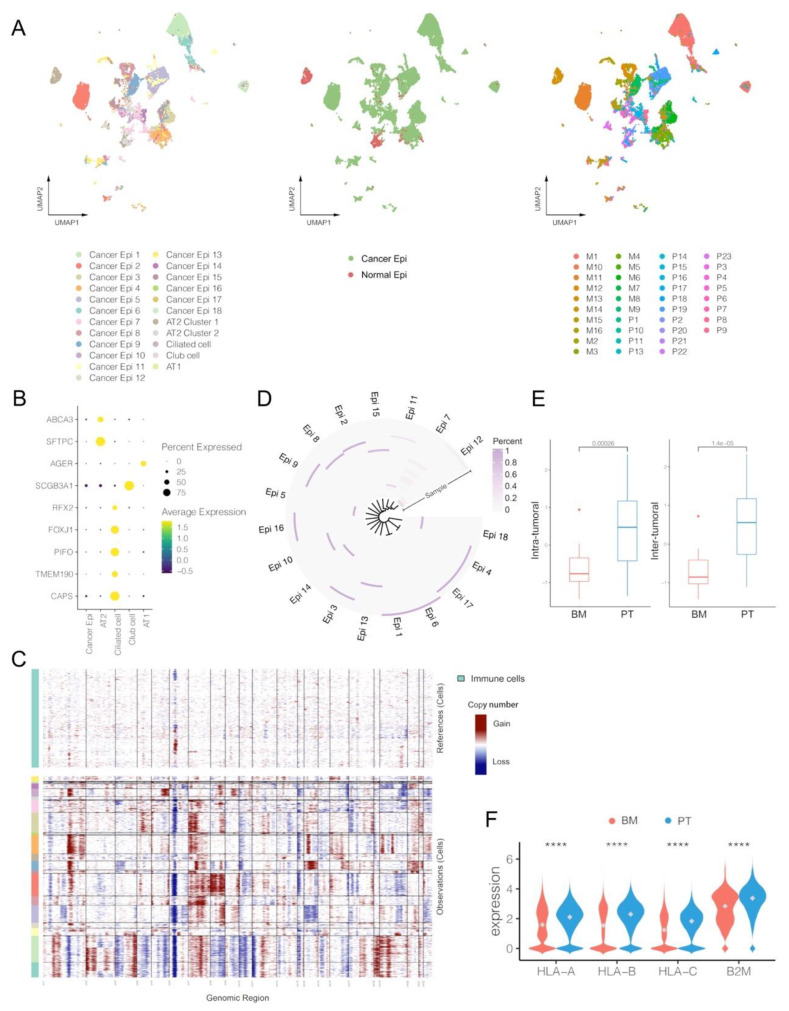
Single-cell characterization of epithelial cells in primary tumors (PT) and brain metastases (BM) of lung adenocarcinomas. (**A**) UMAP plot of epithelial cells by clusters, by cancer vs. normal epithelial, and by patients (M: brain metastasis; P: primary tumor). (**B**) Average expression and cell precent expressed of marker genes in each normal epithelial cluster. (**C**) Estimation of copy number alterations (CNAs) in each cluster of cancer epithelial cells by inferCNV (immune cells were set as references). (**D**) Proportions of cancer epithelial cells originating from each tumor sample in each cancer clusters. (**E**) Comparisons of intra-tumoral and inter-tumoral heterogeneity between PT and BM tumors. (**F**) Expression levels of HLA-I genes between cancer epithelial cells of BM and PT groups (Wilcox rank-sum test, **** *p* value < 0.0001). Alveolar type 1 cells (AT1); Alveolar type 2 cells (AT2).

Epithelial cells were further divided into cancer and normal epithelial cells, but demonstrated great heterogeneity between different tumor samples (Figure 4A and Figure S5A). The normal epithelial cells involved several common types located at normal pulmonary tissues, such as ciliated cells (marked by *RFX2*, *FPXJ1* and *CAPS*), alveolar type 1 (AT1; marked by *AGER*), alveolar type 2 (AT2; marked by *ABCA3* and *SFTPC*) and club cells (marked by SCGB3A1), almost all of which were observed in the PT group (Figure 4B) [9]. The CNA analyses further confirmed that cancer cells demonstrated obvious changes compared to the reference immune cells, but normal epithelial cells did not (Figure 4C). Even among cancer cells, gene expression profiles and cell number distributions greatly varied between samples and clusters, and almost every cancer epithelial cluster came from one single specific tumor sample, indicating prominent inter-tumor heterogeneity (Figure 4D). Moreover, different marker genes were also specifically found in certain cluster (Figure S5A). We also quantified the tumor heterogeneity based on the top hundred principal components of the gene expression matrix. The results suggested that the BM group had significantly lower scores in both inter- and intra-tumoral heterogeneity than PT (*p* < 0.001; Figure 4E), which also had a strong correlation among all samples (Pearson correlation, R = 0.99, *p* < 0.001; Figure S5B). Meanwhile, expression levels of human leukocyte antigen class I (HLA-I)-associated genes (*HLA-A*, *HLA-B*, *HLA-C* and *B2M*) were also found significantly lower in the BM group, suggesting an inferior antigen presentation capacity within TME of BM (Figure 4F). Anyway, the BM tumors presented lower heterogeneity than PT, probably because only a proportion of cancer cells shed from the primary site to successfully form a metastatic tumor, which had been observed in multiple metastatic diseases [35].

### 3.5. Lack of Inflammatory-like CAFs and Enrichment of Pericytes with Ambiguous Phenotypes within BM Tumors

**Figure 5:**
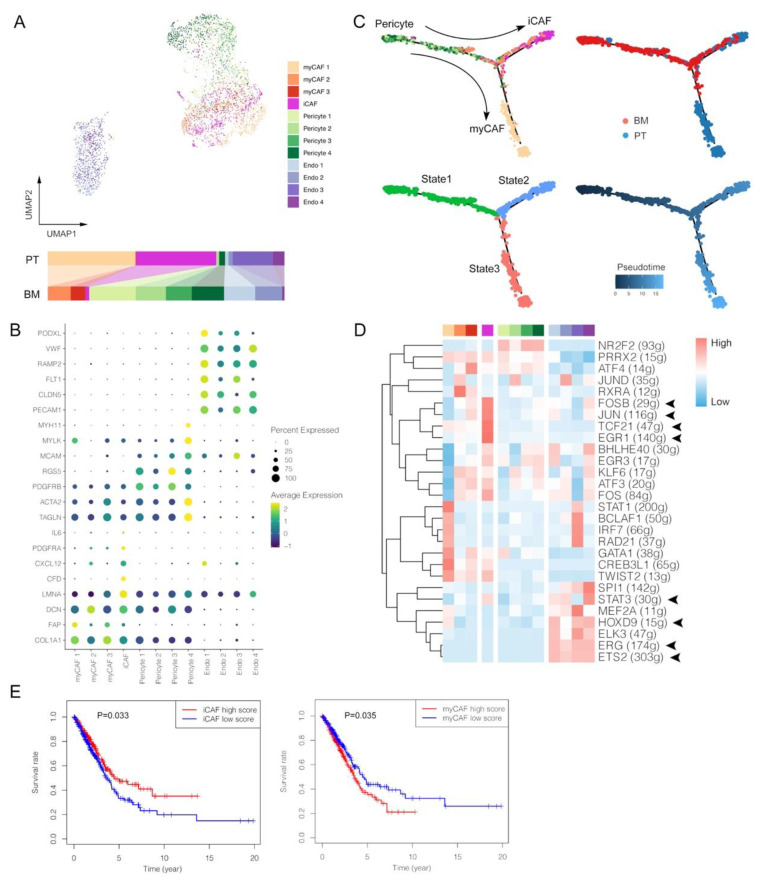
Single-cell characterization of stromal cells in primary tumors (PT) and brain metastases (BM) of lung adenocarcinomas. (**A**) UMAP plot of stromal cells. (**B**) Average expression and cell precent expressed of marker genes in each stromal cell cluster. (**C**) Pseudotime trajectory analysis of pericytes and CAFs. (**D**) Normalized activations of transcription factors in each stromal cell cluster. The number of predicted target genes for each transcription factor was shown in the latter brackets. (**E**) Kaplan–Meier survival curves of iCAF and myCAF signatures in patients with lung adenocarcinomas originating from The Cancer Genome Atlas (TCGA) Program. Inflammatory-like cancer-associated fibroblasts (iCAF); myofibroblast CAF (myCAF); endothelial cells (Endo).

Stromal cells within tumors shapes TME by multiple complex mechanisms [36,37,38]. In our analyses, stromal cells were clustered into myCAF (marked by *ACTA2*, *TAGLN*, *COL1A1* and *FAP*), iCAF (marked by *CFD*, *CXCL12*, *PDGFRA* and *IL6*), pericytes (marked by *MCAM* and *RGS5*) and endothelial cells (marked by *PECAM1*, *VWF* and *RAMP2*) (Figure 5A,B and Figure S6A). Firstly, the distribution of CAF clusters seemed to present some specificity, such as that myCAF1 and iCAF mainly existed in PT, further indicated in distributions by each patient (Figure S6B). The pseudotime analysis showed myCAF and iCAF potentially originated from the same pericytes but via different differentiation trajectories, suggesting the different induction mechanisms in BM and PT (Figure 5C). Based on TF analyses, anti-tumoral iCAF showed enriched activation of cell survival-associated genes, such as *FOSB*, *JUN* and *EGR1* (Figure 5D) [39]. *TCF21*, which can dampen the ability of tumor invasion, chemoresistance and growth in vivo by regulating the CAF state, was also extensively activated in iCAF cells from primary LUAD [40]. Instead, few iCAF cells were observed in the BM group, further indicating an inflammation-limited TME with BM tumors. Among more than five hundred LUAD patients from TCGA database, we assessed enrichment scores of marker gene sets of iCAF and myCAF using ssGSEA, and the results were consistent with observations in our scRNA analyses that iCAF, deficient in BM tumors, was associated with a better survival, while myCAF exactly showed the opposite effect (Figure 5E). Thus, the lack of iCAF may be one of reasons for the poorer outcome of BM patients.

Meanwhile, most of pericytes were found in the BM group, which might be associated with their function in maintaining brain-blood barrier (BBB), supported by the fact that almost every BM tumor involved many pericytes (Figure S6B). Specifically, pericyte cluster 4 can be further regarded as the differentiated subtype that highly expressed functional gene markers as *TAGLN*, *MYLK* and *MYH11* [41]. Nonetheless, the relationship between pericytes and BM remains to be elucidated, since previous studies demonstrated inconsistent viewpoints that pericytes promoted BM in breast cancer and prevented BM in lung cancer in vitro [42,43]. These results of CAF and pericytes might also explain that components within TME of BM tumors remained relatively simple, unlike the complex environment caused by the long-term selection and evolution of primary tumors.

Four clusters of endothelial cells were identified with high expression levels of marker genes, such as *PECAM1* and *VWF*, normally functioning in interacting between endothelia cells and platelets (Figure 5B). PODXL, a gene encoded podocalyxin normally in vascular endothelial cells, was highly expressed in Endo 1, which mainly existed in the BM group (Figure 5A). Overexpression of PODXL within the tumor tissue was associated with poor prognosis, aggressive invasion, chemoresistance and unfavorable therapeutic effects, supporting its role in BM diseases [44]. Though Endo 1 and 2 were predominantly located in BM, and Endo 3 and 4 mainly in PT, several angiogenesis-related TFs were simultaneously activated, including *ETS2*, *ERG*, *HOXD9* and *STAT3*, probably suggesting their pro-tumoral effects (Figure 5D) [45,46,47,48].

### 3.6. Cell-Cell Interactions Indicate the Critical Role of Pericytes in Shaping TME of Brain Metastasis

**Figure 6:**
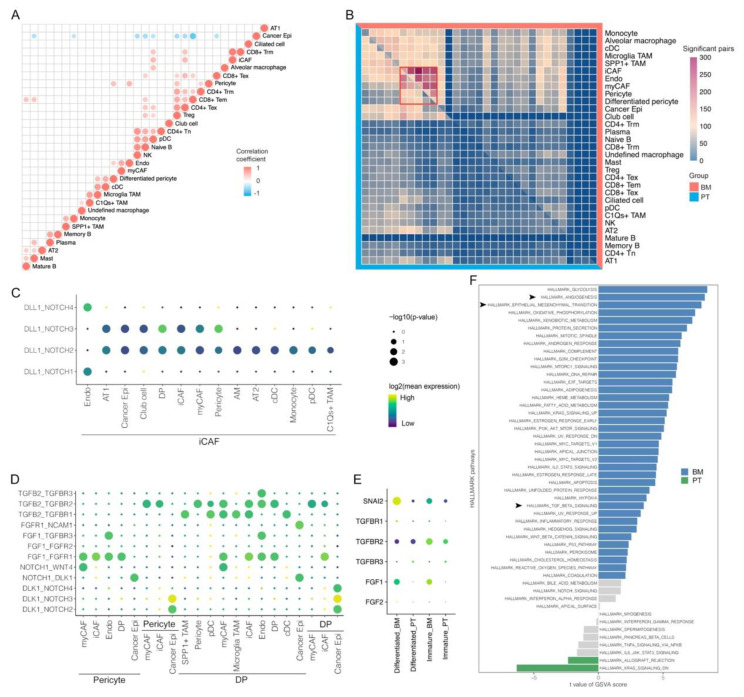
Specific cell-cell interactions in primary tumors (PT) and brain metastases (BM) of lung adenocarcinomas. (**A**) Correlations of cell proportions for each non-epithelial cluster in each tumor sample (Pearson correlation). (**B**) Comparisons of significant cell-cell interactions between BM and PT tumors. (**C**) Significant interaction pairs specifically in PT groups. (**D**) Significant interaction pairs specifically in BM groups. (**E**) Specific gene expressions in differentiated and immature pericytes between BM and PT tumors. (**F**) Activation differences of pathways for pericytes between BM and PT tumors by GSVA analysis. Differentiated pericytes (DP).

In addition to relevant analyses within each cell cluster, cell–cell interactions between clusters are critical in shaping tumor immune microenvironment. Proportion differences for each cell type were compared, and the results found that most immune cells were higher in the PT group (Figure S6). As mentioned before, for example, functional CD8+ Trm cells were much fewer in the BM group, indicating the lack of anti-cancer immunity within BM tumors. Furthermore, correlations of cell contents between cell types were also explored in Figure 6A. Cancer cells had only negative associations with certain cells, including pericytes, CD8+ Tem, CD4+ Tex, Treg, pDC, naïve B cells, NK cells, monocytes and mast cells. Most of them were common cells within tumors and generally accounted for relatively large percentages among non-cancer cell populations, thus normally presenting negative associations. However, pericytes that were enriched in the BM group, may have specific interactions with cancer cells [49,50,51].

The CellPhoneDB method was used to evaluate cell–cell interactions between cell subtypes. We firstly calculated the number of ligand-receptor pairs with significant interactions between cell types, and observed that they were enriched among iCAF, endothelial cells, myCAF and pericytes, suggesting their potentials in shaping TME, especially in the BM group (Figure 6B). In the PT group, Delta Like Canonical Notch Ligand 1 (DLL1)-mediated canonical NOTCH pathways were universally activated via interactions between iCAF and many other cells, which corresponded to its favorable role in anti-cancer immunity in lung cancer [52,53,54], while instead, there were enriched activations of noncanonical NOTCH pathways via interactions of Delta-like Noncanonical Notch Ligand 1 (DLK1)-NOTCH between cancer cells and pericytes (the immature type for *NOTCH1*, *NOTCH2* and *NOTCH3*; the differentiated type for *NOTCH1*, *NOTCH2*, *NOTCH3* and *NOTCH4*) in the BM groups (Figure 6C). Accumulative evidence has demonstrated that increased expression of DLK1 was observed with disease progression and negative prognostic effects in numerous malignancies [55,56,57], and could also recover and promote stemness of tumor cells [58]. However, the actual relationship between DLK-1 and NOTCH signaling has not been clarified clearly with conflicting results from previous studies in distinct models [59].

We also identified widespread interactions of TGFβ-TGFβR between pericytes and other cells within TME, especially differentiated pericytes (Figure 6D). Normally, ericytes are critical in regulating angiogenesis and maintaining BBB integrity [60]. In brain tumors, cancer cells can induce phenotype transition of pericytes form the tumor-suppressive to the immature type, thus initiating tumor progression [61,62], consistent with our results that most pericytes within BM tumors demonstrated immature signatures. Besides, TGFβ within TME modulated function of the surrounding pericytes, and promoted cell proliferation, migration and morphological changes for vascular proliferation via upregulating downstream epithelial-mesenchymal transition (EMT)-related factors [63], such as SLUG, also called Snail Family Transcriptional Repressor 2, encoded by *SNAI2*, which showed significantly higher expression levels in pericytes within BM (Figure 6E). In accordance with the study in the glioblastoma model from Wirsik et al., we also identified TGFBR2 as the most prominent receptor that participated in activation of the TGFβ signaling pathway in pericytes [63]. Furthermore, it was verified in the GSVA analysis that pericytes within BM tumors had significantly enriched activations of angiogenesis, EMT and TGFβ signaling pathways when compared with those within PT tumors (Figure 6F). Thus, we hypothesize that TGFβ released by various types of cells within TME may interact with TGFBR2 in abundant pericytes within BM tumors to modulate cancer angiogenesis. Meanwhile, it was reported that cancer cell–CAF (iCAF and myCAF) crosstalk could enhance tumor vascularization by promoting TGFβ-mediated pericyte-endothelial cell interactions [64]. These results may suggest the critical role of interactions between pericytes and other cells within TME in the formation of unstructured BM vasculature, which shapes immune cell desertification and BBB breakdown in BM tumors [65].

### 3.7. Pericyte-Related Machine Learning Models Predicting Responses to ICI Therapy

**Figure 7:**
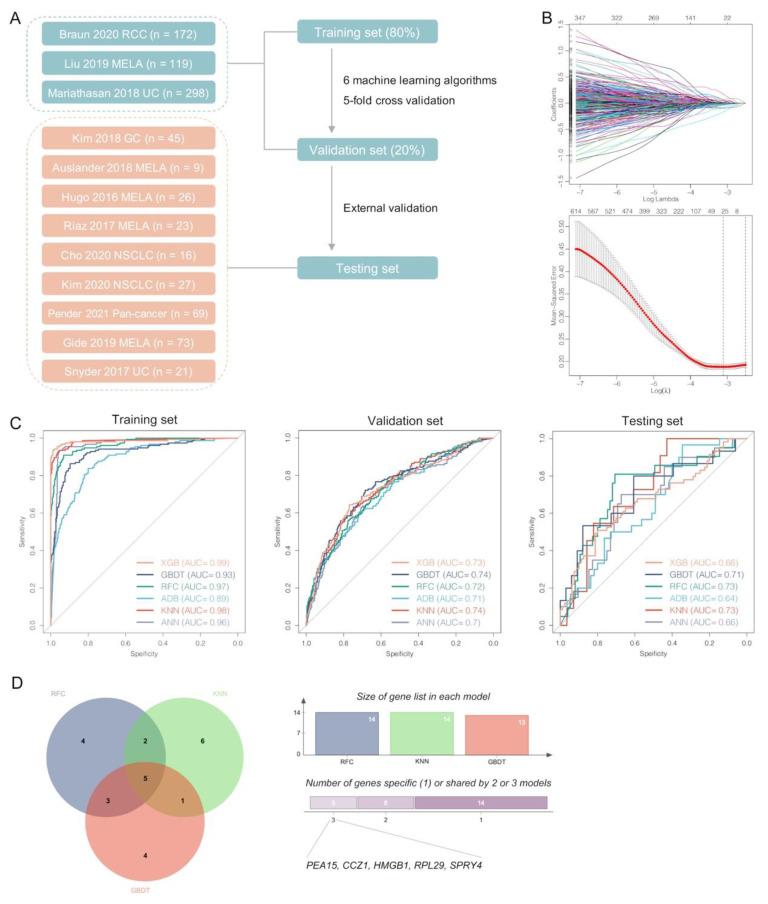
Development and validation of multiple machine learning-based models predicting responses to immunotherapy with pericyte-related differentially expressed genes. (**A**) Schematic diagram of predictive models by six machine learning algorithms using the previous cohorts of cancer immunotherapy. (**B**) LASSO regression for gene selection. (**C**) ROC curves of machine learning models in the training, validation and testing set. (**D**) Venn diagram for three optimal models (RFC, GBDT and KNN) that identified the most potential pericyte-related genes in predicting immunotherapeutic responses.

To better validate the importance of pericytes in ICI therapy, a total of 4,138 differentially expressed genes were identified between pericytes and other stromal cell clusters at the start. Using the previous ICI therapy cohorts (Figure 7A), we further found 32 genes contributed most to the related therapeutic responses by LASSO regression (Figure 7B). Next, six classical machine learning algorithms were applied to develop predictive models for responses to ICI therapy with five-fold cross validation. All of these models demonstrated great performance in the training (AUC: 0.89–0.99) and validation sets (AUC: 0.70–0.74), though with different gene lists for modeling (Figure 7C and Table S1). In the external test set, three models (GBDT, RFC and KNN; AUC: 0.71–0.73) performed obviously than others (XGB, ADB and ANN; AUC: 0.64–0.66). The five common genes used in GBDT, RFC and KNN models were *PEA15*, *CCZ1*, *HMGB1*, *RPL29* and *SPRY4* (expression levels seen in Figure S7), which may be critical to pericytes’ interactions with TME and can be potential targets to improve immunotherapeutic efficacy in LUAD patients with brain metastasis (Figure 7D). For example, beside extensively engaging in tumor development and progression, *HMGB1* indeed had profound effects in remodeling TME, and its inhibition could enhance ICI efficacy [66].

## 4. Discussion

In the present study, we displayed the comprehensive single-cell landscape of primary advanced LUAD and brain metastases by integrated analyses of scRNA-seq data from 39 tumor specimens. We revealed the characteristic heterogeneity of cells and microenvironments within tumors of PT and BM, and further analyzed potential variations in cellular and molecular programs shaping metastatic progression from primary sites to the brain. In the valuable atlas, three major cell components were identified that involved a total of 49 subtypes besides 18 clusters of cancer epithelial cells, which demonstrated specific inter-patient heterogeneity. To our knowledge, this study also provided a largest comparative analysis between primary LUAD and brain metastases at the single-cell level.

In total, BM tumors showed a higher percentage of cancer cells, but with fewer infiltrations of immune cells and stromal cells than the primary LUAD. In particular, T cell subclusters might be specific to the two groups of tumors. CD8+ Trm is critical in anti-cancer immunity, but was found deleted within BM tumors. Moreover, CD8+ Tem cells in the BM group demonstrated more exhausted status than PT. GO analyses also confirmed the obvious T cell activation of CD8+ Trm rather than Tem, and pro-inflammatory TFs were specifically enriched in the CD8+ Trm cluster. Based on the factors mentioned above, we hypothesize that BM tumors may present a dysfunctional phenotype of adaptive immunity for CD8+ Trm desertification.

Two different TAM clusters, C1Qs+ and SPP1+ TAMs, were identified, which also demonstrated certain specificity to the BM and PT groups, further supported by their distinct differentiation trajectories from monocytes in the pseudotime analysis. C1Qs+ TAM, mainly located in BM, highly expressed complement C1q-related genes (such as C1QB, C1QA, C1QC), promoting phagocytosis and anti-inflammatory function within TME [34]. Besides, we also identified one cluster of alveolar macrophages in PT and one cluster of microglia TAMs in BM, which presented distinct phenotypes. Therefore, variations of multiple macrophages may engage in shaping the more unfavorable TME within BM tumors.

By analyzing epithelial marker genes and CNA, we confirmed the normal epithelial cell and the cancer epithelial cell, while the former was nearly sourced from the PT group. We also observed significantly lower inter- and intra-tumoral heterogeneity and the inferior expression level of HLA-I genes in BM than PT. Similarly, iCAF cells were found lacking in the BM group, which can contribute to a more inflammatory TME. However, the unfavorable myCAF existed within BM tumors, demonstrating different developmental trajectories from the myCAF. The validation tests using TCGA database presented the two types of CAF indeed had opposite effects on survival. Therefore, we thought the deletion of iCAF within TME of BM might be one of reasons that caused worse outcome in BM patients. Overall, based on our comparative results, there may be more simple components within TME of BM tumors, considering that the metastatic lesions formed by only a proportion of circulating cancer cells shed from the primary tumors, and thus did not present a complex tumor environment like primary tumors with having encountered long-term selection and evolution.

Based on cell–cell interaction analysis, we propose a potential mechanism that may be responsible for unstructured tumor vasculature in BM. Since pericytes were mainly found in BM tumors, they may have widespread interactions of TGFβ-TGFBR2 with various types of cells to regulate cancer vascularization via increased expression of SLUG. Abnormal angiogenesis within BM tumors can adversely affect immune cell infiltration and contribute to breakdown of BBB to promote tumor metastasis [65]. Furthermore, using previous immunotherapy cohorts, we developed and validated six machine learning-based models predicting responses to ICI therapy in multiple cancers based on pericyte-specific genes. These models showed great predictive performance, highlighting the critical roles of pericytes in shaping TME of brain metastasis among LUAD patients.

## 5. Conclusions

Overall, as the most comprehensive comparative analyses of LUAD and brain metastases at the single-cell resolution, our study may provide new insights into the TME heterogeneity between PT and BM. We also identified a lack of several specific cell clusters that might be responsible for the worse outcome in BM patients and further explored their potential mechanisms by multiple analyses. Pericytes may be one of the important cell clusters in the TME of brain metastasis, and we also identified several critical genes associated with pericytes for optimal prediction of ICI therapy responses, which can be potential targets to improve immunotherapeutic efficacy.

## Data Availability

The data involved in this study are available online at GEO database under accession number GSE148071, GSE131907, GSE186344 and GSE143423 and TCGA database.

## Supplementary Materials

The following supporting information can be downloaded at: https://www.mdpi.com/article/10.3390/biom13010185/s1, Figure S1: (A) Quality control of single-cell data. Red lines indicate cut-off values for relevant filtering processes. (B) Feature plot of marker gene expressions for identification of immune, stromal and epithelial cells. (C) Feature plot of marker gene expressions for identification of immune cell subclusters; Figure S2: (A) UMAP plot colored by individual patient. (B) UMAP plot colored by primary tumors and brain metastasis of lung adenocarcinomas. (C) Proportions of epithelial, immune and stromal cells in each patient. (D) Proportions of each immune cell subcluster in each patient. (E) Cell number of immune cell clusters per 10,000 cells between primary tumors and brain metastasis of lung adenocarcinomas; Figure S3: (A) Top enriched biological process terms in CD8+ T clusters by GO analysis. (B) Correlation between percentages of Treg and CD4+ Tex within each tumor in primary tumors (PT) and brain metastasis (BM) of lung adenocarcinomas. (C) Signature scores of gene sets associated with naïveness, cytotoxicity, exhaustion, proliferation, and Treg of each T cell cluster. (D) Signature scores of gene sets in CD4+ T between PT and BM. (E) Signature scores of gene sets in CD8+ T between PT and BM. (F) Correlation between exhaustion score and pseudotime in CD8+ Tem (Pearson correlation). (G) UMAP plot of natural killer (NK) cells. (H) Average expression and cell precent expressed of marker genes in each NK cell cluster. (I) Comparisons of signature scores of NK cells between PT and BM. CD4+ naïve T cell (CD4+ Tn), CD4+ memory T cell (CD4+ Tm), CD4+ exhausted T cell (CD4+ Tex), Treg, CD8+ tissue-resident memory T cell (CD8+ Trm), CD8+ effector memory T cell (CD8+ Tem), CD8+ exhausted T cell (CD8+ Tex), * *p* value < 0.05, ** *p* value < 0.01, *** *p* value < 0.001, **** *p* value < 0.0001; Figure S4: (A) Heatmap of differentially expressed genes for each myeloid cell cluster. (B) Correlations between scores of activation and migration/tolerance among classical dendritic cells (Pearson correlation); Figure S5: (A) Heatmap of differentially expressed genes for each epithelial cell cluster. (B) Correlation between intra-tumoral and inter-tumoral heterogeneity of cancer epithelial cells (Pearson correlation) in primary tumors (PT) and brain metastases (BM) of lung adenocarcinomas; Figure S6: (A) Heatmap of differentially expressed genes for each stromal cell cluster. (B) Proportions of each stromal cell subcluster in each patient; Figure S7: Expression levels of five candidate genes within pericytes between primary tumors (PT) and brain metastases (BM) of lung adenocarcinomas for prediction of immunotherapeutic responses (Wilcox test); Table S1: Identified genes for development of machine learning models.
