# Supplementary figures and images for: Single-Cell Profiling Comparisons of Tumor Microenvironment between Primary Advanced Lung Adenocarcinomas and Brain Metastases and Machine Learning Algorithms in Predicting Immunotherapeutic Responses
Source: Biomolecules. 2023 Jan 16;13(1):185. doi: 10.3390/biom13010185 (PMC9855438; doi:10.3390/biom13010185)

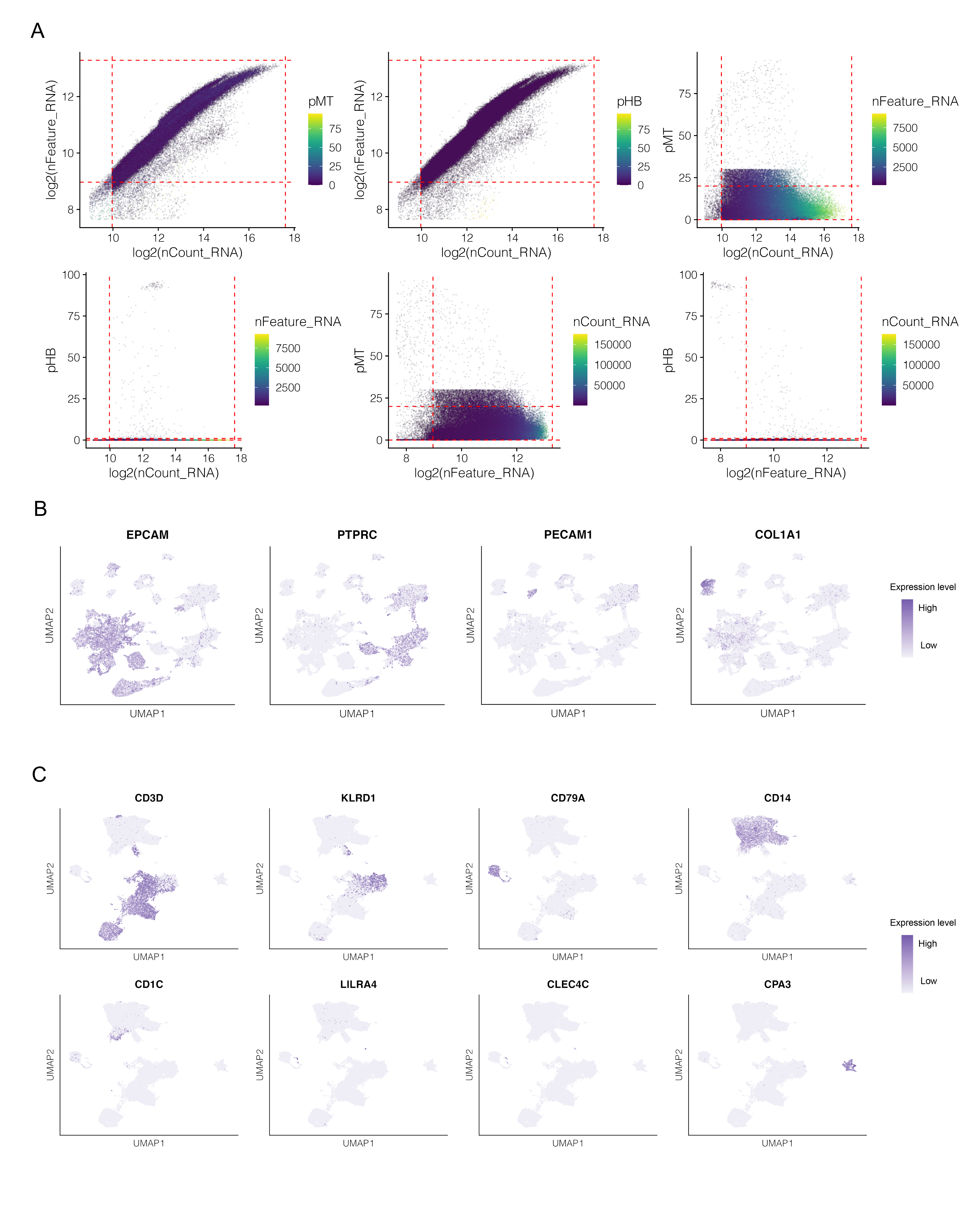

Supplement: Supplementary file 1 [file biomolecules-13-00185-s001.zip › Figure S1.jpg]

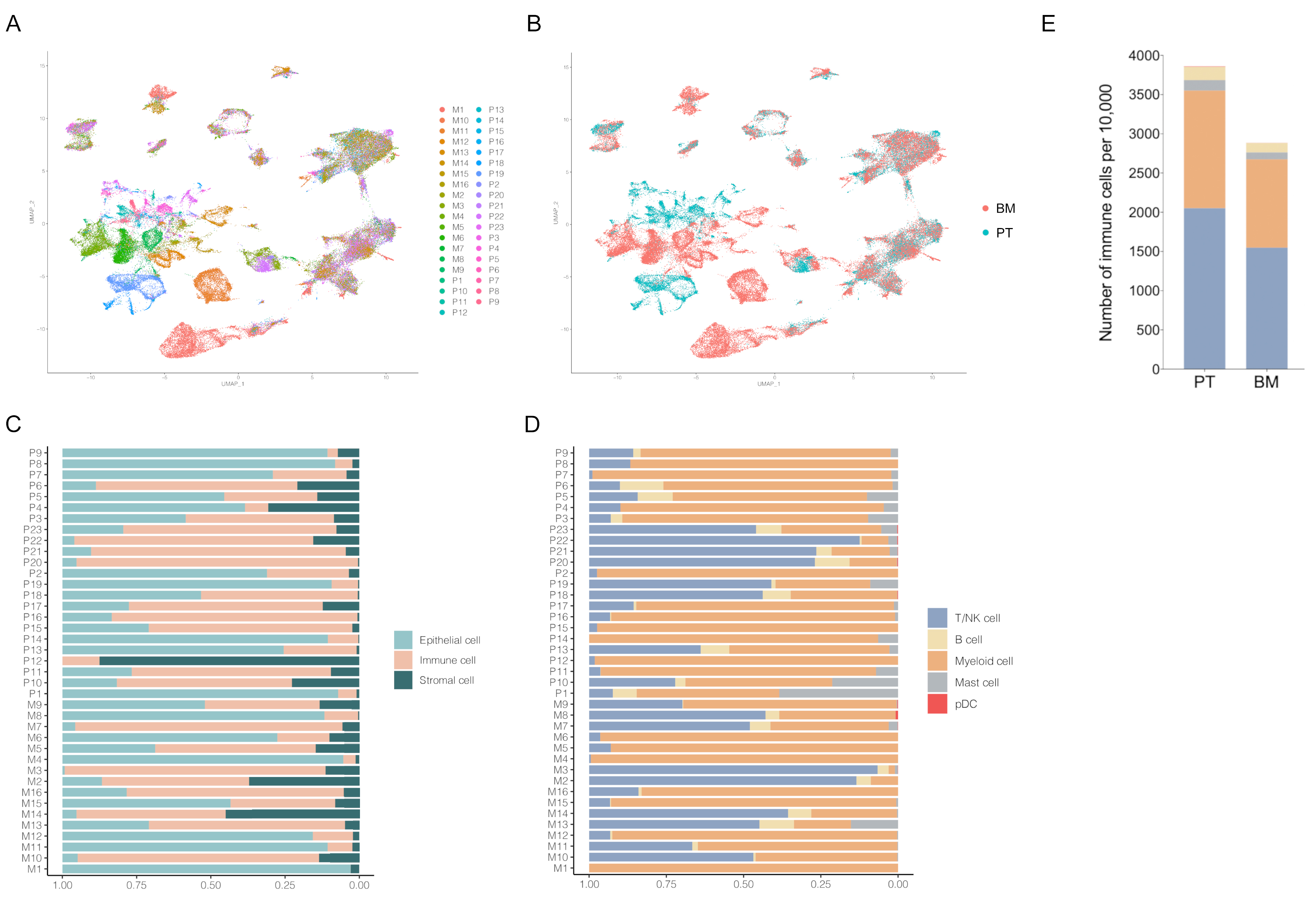

Supplement: Supplementary file 1 [file biomolecules-13-00185-s001.zip › Figure S2.jpg]

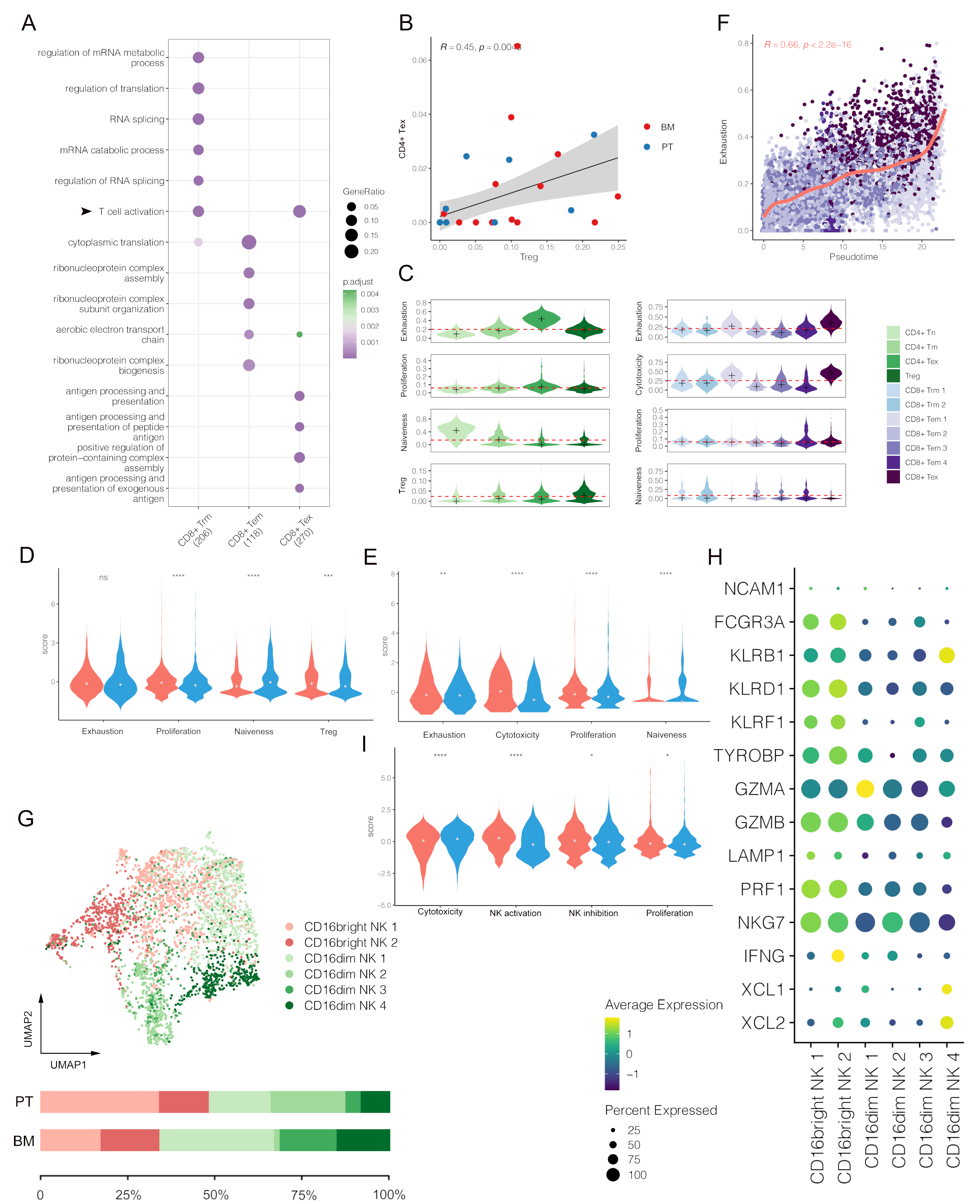

Supplement: Supplementary file 1 [file biomolecules-13-00185-s001.zip › Figure S3.jpg]

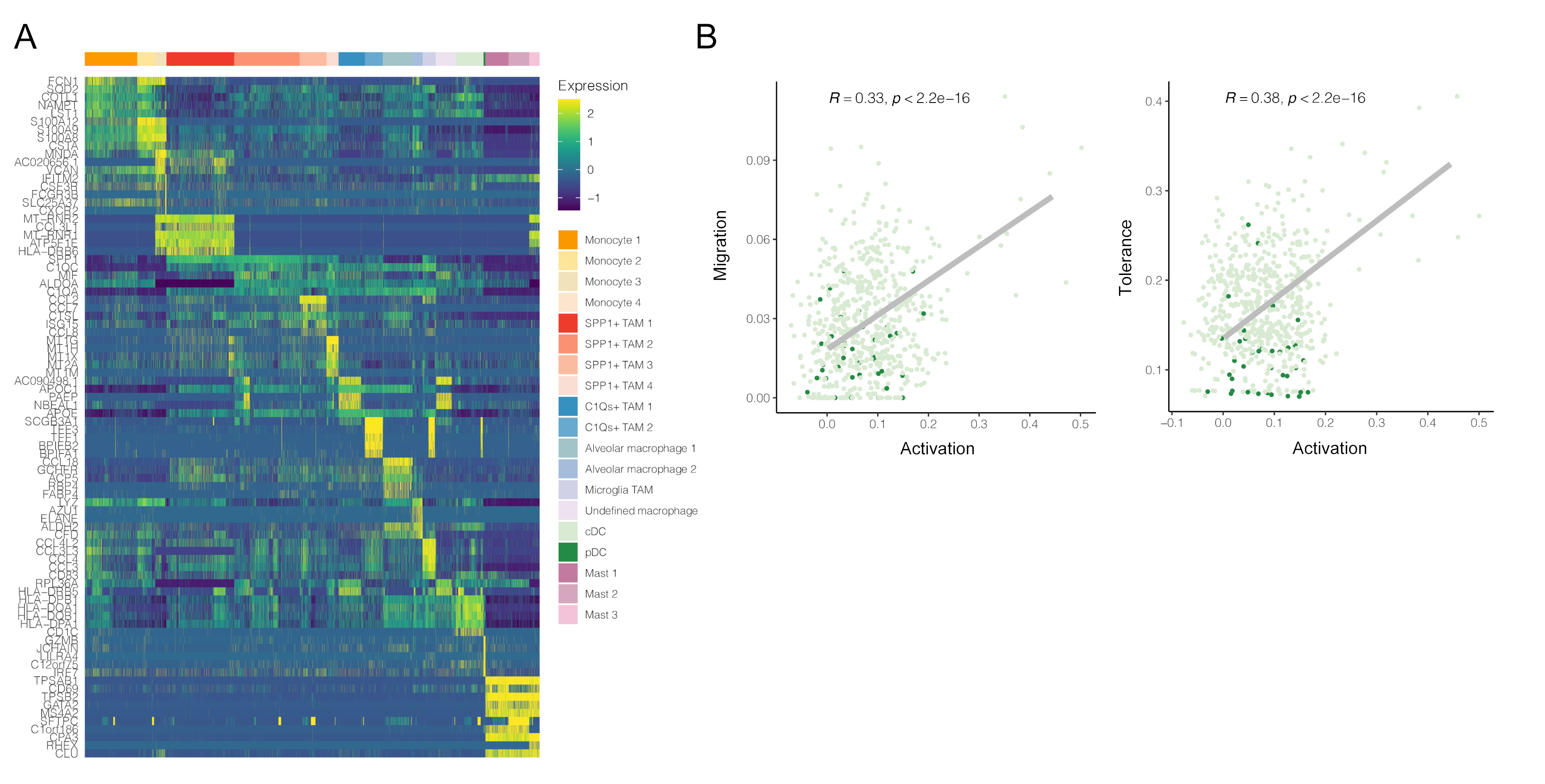

Supplement: Supplementary file 1 [file biomolecules-13-00185-s001.zip › Figure S4.jpg]

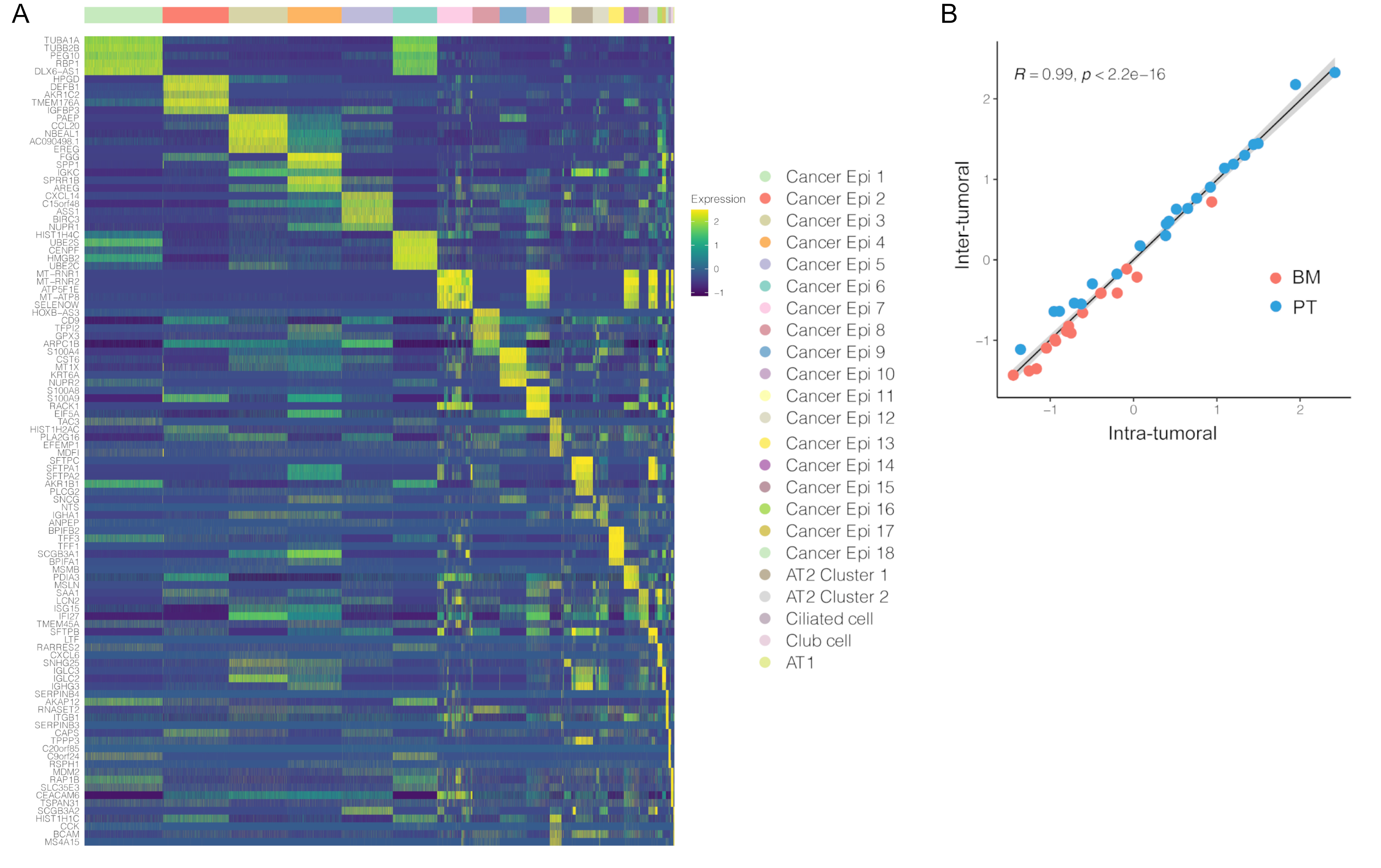

Supplement: Supplementary file 1 [file biomolecules-13-00185-s001.zip › Figure S5.jpg]

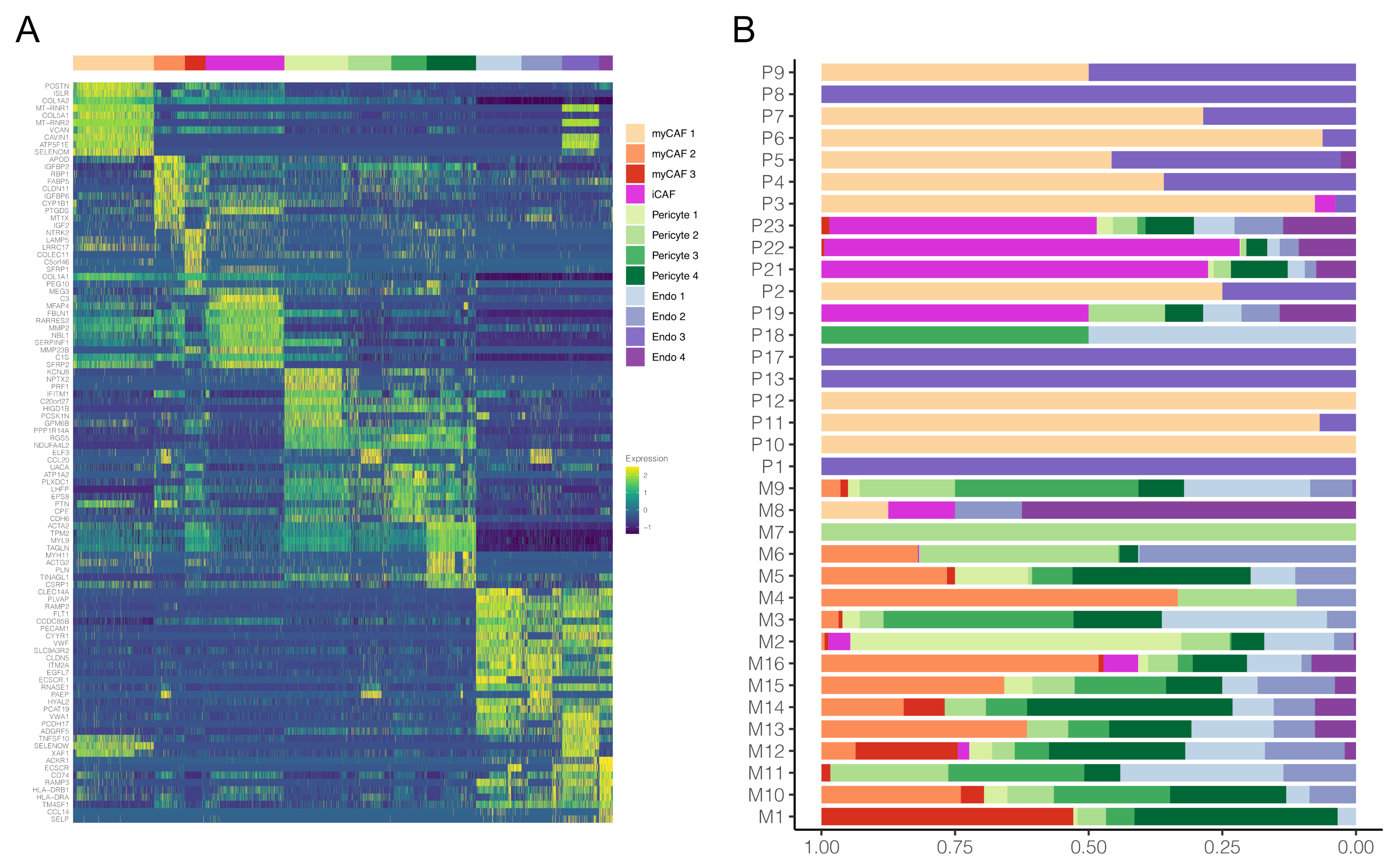

Supplement: Supplementary file 1 [file biomolecules-13-00185-s001.zip › Figure S6.jpg]

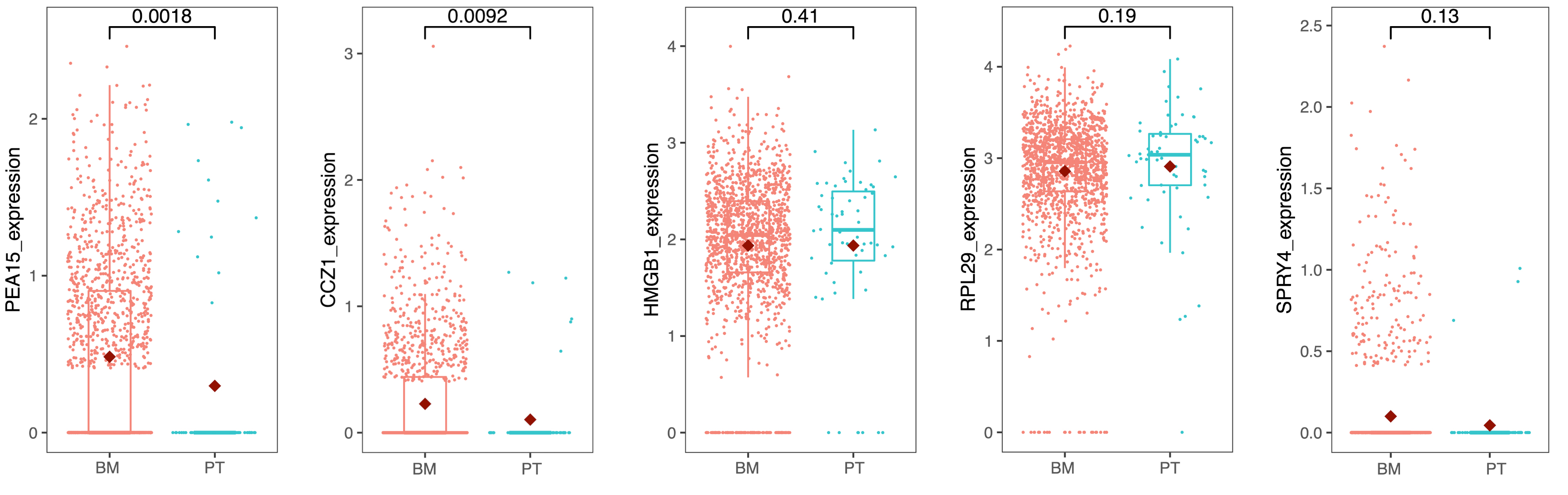

Supplement: Supplementary file 1 [file biomolecules-13-00185-s001.zip › Figure S7.jpg]
